# Ethylic Esters as Green Solvents for the Extraction of Intracellular Polyhydroxyalkanoates Produced by Mixed Microbial Culture

**DOI:** 10.3390/polym13162789

**Published:** 2021-08-19

**Authors:** Sara Alfano, Laura Lorini, Mauro Majone, Fabio Sciubba, Francesco Valentino, Andrea Martinelli

**Affiliations:** 1 Department of Chemistry, Sapienza University of Rome, P.le Aldo Moro 5, 00185 Rome, Italy; sara.alfano@uniroma1.it (S.A.); laura.lorini@uniroma1.it (L.L.); mauro.majone@uniroma1.it (M.M.); 2 NMR-Based Metabolomics Laboratory (NMLab), Sapienza University of Rome, P.le Aldo Moro 5, 00185 Rome, Italy; fabio.sciubba@uniroma1.it; 3 Department of Environmental Sciences, Informatics and Statistics, Ca Foscari University of Venice, Via Torino 155, 30170 Mestre-Venice, Italy; francesco.valentino@unive.it

**Keywords:** polyhydroxyalkanoates, molecular weight, downstream processing, ethyl esters, mixed microbial culture, PHA extraction

## Abstract

Volatile fatty acids obtained from the fermentation of the organic fraction of municipal solid waste can be used as raw materials for non-toxic ethyl ester (EE) synthesis as well as feedstock for the production of polyhydroxyalkanoates (PHAs). Taking advantage of the concept of an integrated process of a bio-refinery, in the present paper, a systematic investigation on the extraction of intracellular poly(3-hydroxybutyrate-*co*-3-hydroxyvalerate), produced by mixed microbial culture by using EEs was reported. Among the tested EEs, ethyl acetate (EA) was the best solvent, dissolving the copolymer at the lowest temperature. Then, extraction experiments were carried out by EA at different temperatures on two biomass samples containing PHAs with different average molecular weights. The parallel characterization of the extracted and non-extracted PHAs evidenced that at the lower temperature (100 °C) EA solubilizes preferentially the polymer fractions richer in 3HV comonomers and with the lower molecular weight. By increasing the extraction temperature from 100 °C to 125 °C, an increase of recovery from about 50 to 80 wt% and a molecular weight reduction from 48% to 65% was observed. The results highlighted that the extracted polymer purity is always above 90 wt% and that it is possible to choose the proper extraction condition to maximize the recovery yield at the expense of polymer fractionation and degradation at high temperatures or use milder conditions to maintain the original properties of a polymer.

## 1. Introduction

Stimulated by environmental concerns, the great demand for biodegradable and compostable plastic materials is partially hampered by economic reasons. In fact, although suitable alternatives to oil-based commodity polymers are available, the cost to produce bio-based polymers is higher than that of conventional plastics [1]. Polyhydroxyalkanoates (PHAs) are a class of biodegradable polyesters that can be obtained from renewable sources and, according to the composition and processing conditions, have suitable properties able to replace in some applications widespread materials, like polyethylene or polypropylene. Poly(3-hydroxybutyrate) [P(3HB)] and its copolymers, mainly with 3-hydroxyvalerate comonomeric repeating unit [P(3HB-*co*-3HV)], are among the most investigated biopolymers of this class and are produced at an industrial scale [2]. The PHAs are biosynthesized as storage material by a wide range of bacteria and various consolidated strategies have been applied to maximize the accumulation of the polymer into the cells. However, the cost of the polymer is not yet competitive and, at the moment, is exploited in high added value applications, including biomedical devices, or when specific regulations require degradable and compostable goods. For instance, PHAs can be used in sustainable packaging applications replacing conventional petrochemical products [3,4] and processed as films, fibers and foams for everyday articles such as shampoo bottles and plastic beverage bottles due to their renewability, biodegradability and high water vapor barrier [5]. Three are the main factors involved in the high PHA production cost. In order to assure a stable production and chemical composition as well as chemical–physical properties of the product, pure microbial cultures and a careful selection of the feedstock are necessary. This implies expensive sterilized conditions of fermentation reactor and substrates. The extraction and purification of the polymer from non-PHA cell mass (NPCM) is a further factor contributing to a large part of the production cost [6].

Many attempts have been investigated and experimented with to overcome these drawbacks and alternative production processes have reached the pilot plant scale [7,8,9,10,11,12]. They are mainly based on the use of mixed microbial culture (MMC) production in which the selection of microorganisms with high PHA storage capacity and biomass enrichment is obtained by aerobic dynamic feeding conditions (ADF) through the well-known feast–famine regime [13]. As a result, the use of MMC allowed to feed microorganisms with volatile fatty acid (VFA) obtained from the anaerobic fermentation of waste organic fractions of different origins rather than more expensive VFA synthetic mixtures [14,15]. Then, the MMC-based process could be a suitable cheaper alternative to the pure culture PHA production, especially taking into account that the polymers obtained from the two processes show comparable thermal and mechanical properties, mostly affecting the composition and molecular weight (MW) [16].

As far as the final downstream production process, polymer separation and recovery from the non-polymer cellular material is mainly carried out by two methods, namely disruption of NPCM through oxidation by sodium hypochlorite, hydrogen peroxide or by concentrated alkaline water solution, possibly favored by surfactants, or dissolution of PHA in an appropriate organic solvent [17].

The latter is an attractive procedure widely investigated, as evidenced by the great number of scientific papers, reviews or patents on this topic, mainly dealing with PHAs from pure culture [17,18,19,20,21,22,23,24]. In alternative to chlorinated hydrocarbons, which are the best solvents for PHAs, many research studies address the use of green solvents, with low or no toxicity and possibly derived from biochemical conversion, to overcome ecological issues, limitations involving worker safety or stringent regulations on solvent traces in goods for particular applications [25]. Solvents characterized by low toxicity, including ethers [26,27], esters [25,27,28,29], carbonates [29,30,31,32] and ketones [28,33,34], have been identified as appealing alternatives to chlorinated hydrocarbons. Their suitability is evaluated by taking into account their recyclability, the need for biomass pretreatment, polymer recovery yield, the quality of the extracted polymer in terms of purity and possible molecular weight reduction, as well as process cost and environmental performances [35,36].

After the extraction, the dry PHA could be obtained by low boiling point solvent distillation and recovery or by polymer precipitation by an anti-solvent. This latter operation, which could lead to an increase of the PHA purity, is mandatory if the extraction solvent has a high boiling temperature but, on the other hand, make it harder and more expensive the liquid separation and recovery. 

In the present paper, taking advantage of the concept of the integrated process of a bio-refinery, the possible extraction of PHA from biomass by solubilization in ethyl esters of VFA (EEs) was investigated. The EEs, in fact, can be synthetized from the fermentation products of the organic fraction of municipal solid waste (OFMSW) and sewage sludge (SS), the same feedstock used for the production of PHA by mixed culture microorganisms [11,37]. Moreover, the selected EEs, ethyl acetate (EA), ethyl propionate (EP) and ethyl butyrate (EB), have very low toxicity. Together with butyl acetate, EA is also a suggested solvent according to all the solvent selection guides, which attribute low health and environmental scores among the whole ester class [38,39]. Nevertheless, few are scientific papers dealing with the use of EA for the extraction of PHA from single microbial culture and even fewer are those related to the MMC system and, to the best of authors’ knowledge, no literature data are available on the use of EP and EB.

Therefore, because of the appealing possibility to use ethylic esters for the extraction of PHA from biomasses, it was considered useful to carry out a systematic analysis of the process which takes into account the effects of molecular weight of PHA in the biomass and of the extraction conditions on recovery yields, possible polymer fractionation according to PHA composition and molecular weight as well as on the properties of the non-extracted polymer remaining in the biomass. Moreover, the reported outcomes could shed some light on contradictory results reported in the literature.

Then, in this study, EEs at temperatures close or above their boiling points were used to extract PHA from two biomasses produced in a pilot plant working with MMC and a fermented mixture of OFMSW-SS as feedstock [10,11]. In order to investigate the influence of PHA molecular weight on the extractions, one type of biomass was stabilized by thermal treatment, which brings about an MW reduction, while the other by the addition of H_2_SO_4_, which has a small or null influence on MW [40]. Once EA was selected in preliminary experiments as the best solvent among EEs, the extraction with EA was carried out at different temperatures. The chemical and physical–chemical properties, including purity, composition and molecular weight of the extracted PHA as well as of the residual polymer fraction remaining in the biomass, were determined. FTIR and NMR spectroscopy were employed to investigate the cause of the polymer MW reduction observed at the highest extraction temperature.

## 2. Materials and Methods

### 2.1. PHA Production

Within the pilot platform of Treviso (northeast Italy, in the context of a full-scale municipal wastewater treatment plant), the PHA was produced from a feedstock composed of a mixture of (a) the liquid slurry coming from squeezing of the OFMSW and (b) SS from the treatment of urban wastewater. The main process setup (extensively described by Valentino et al., 2019 [10]) comprised of a first anaerobic fermentation reactor (380 L) for PHA-precursors production (volatile fatty acid, VFA), a second aerobic reactor (sequencing batch reactor, SBR; 100 L) for biomass cultivation, and a third fed-batch aerobic reactor (70–90 L) for PHA accumulation within a cellular wall (40–50 wt%). Biomass from two different batches (Biomass1 and Biomass2), with PHA content more than 50% *w*/*w* on dry weight, have been selected to perform polymer extraction and characterization. At the end of accumulation, the two biomasses were centrifuged and stabilized by two different procedures: Biomass1 was subjected to thermal treatment at 145 °C for 30 min followed by overnight drying at 70 °C; Biomass2 was acidified with H_2_SO_4_ down to pH 2.0. Before the extraction, Biomass2 was washed with a 0.3 N NaOH solution up to neutral pH and dried. This step was necessary to avoid polymer acidic hydrolysis by concentrated H_2_SO_4_.

### 2.2. Preliminary Dissolution Tests

Before the extraction experiments, the solvent properties of selected ethylic esters (EEs), were investigated by using reference PHA samples (R-PHA), obtained from the two dry biomasses by continuous chloroform Soxhlet extraction for 8 h. This procedure brings about a nearly complete extraction of the polymer, which maintains unvaried the MW respect that of PHA inside the cells. The dissolution tests were carried out on R-PHAs by employing ethyl acetate (EA, boiling point 77 °C), ethyl propionate (EP, boiling point 102 °C) and ethyl butyrate (EB, boiling point 126 °C), all purchased from Sigma-Aldrich (Sigma-Aldrich, Milan, Italy). An amount of 100 mg of dried R-PHA was placed in a 15 mL thick wall glass pressure vessel with PTFE bushing and Viton O-ring, magnetic stirrer and 4 mL of each ethylic ester. The tube was tightly closed and placed in a silicon-oil bath, preheated at the desired dissolution temperature (T_s_), from 100 to 150 °C. It is important to take care of this procedure because high pressure could be reached into the test tube (P_max_ = 7 atm with EA at 150 °C). The minimum solubilization temperatures (T_Smin_) of R-PHAs were defined as the lowest temperature at which the solution became transparent within 1 h. At the end of the test, each R-PHA sample was vacuum dried at 50–70 °C to a constant weight and the average viscosity molecular weight was determined. For sake of comparison, the dissolution tests were carried out also on a commercial P(3HB) homopolymer (Biomer).

### 2.3. Extraction Experiments

The extraction capacity of the three EEs was evaluated on Biomass1. About 3 g of biomass and 50 mL of EE were inserted in a stainless-steel high pressure stirred mini reactor (Parr 4560, Moline, IL, US), preheated at 70 °C. Then, the reactor was rapidly closed and heated at the extraction temperature T_E_ equal to the minimum solubilization temperature T_Smin_, found in the abovementioned preliminary dissolution tests. After 1 h under stirring, the liquid phase was withdrawn from a dip tube endowed with a stainless still mesh to avoid biomass leakage. At room temperature, the polymer precipitated forming a stable physical gel, which was vacuum dried at 50–70 °C. The obtained solid (extracted PHA, E-PHA) was weighted and characterized by composition analysis and molecular weight determination.

Other than T_E_ = 100 °C, the extraction of PHA from Biomass1 with EA was carried out also at increasing extraction temperatures of 115, 125, 135 and 150 °C. In the experiment carried out at T_E_ = 125 °C, a second extraction on already extracted Biomass1 was performed in the same conditions.

After the recovery of PHA solution in the three EEs, the biomass was vacuum dried and weighted. The residual non-extracted PHA that remained in the biomass (NE-PHA) was solubilized in chloroform by Soxhlet extraction.

The PHA weight fraction recovered by EE extraction (recovery yield, *f_E_*) was evaluated by Equation (1):

(1) $$f_{E}=\frac{w_{s}\times p}{w_{b}\times f_{i}}\frac{V_{0}}{V_{r}}\times 100$$

where *w_s_* and *p* are the weight and purity of solid fraction in the withdrawal, *w_b_* is the weight of the biomass and *f_i_* the initial content of PHA in biomass, *V*_0_ and *V_r_* are the added and recovered liquid volume, respectively. *p* and *f_i_* were evaluated by the gas-chromatographic method (GC).

The weight fraction (*f_NE_*) and the polymer composition (3HB mol%) of non-extracted PHA were quantified by GC. The non-extracted PHA fraction (*f_NE_*) was calculated by taking into account the amount of polymer in non-withdrawal solvent (*w_s_* × [*V*_0_−*V_r_*]), according to Equation (2):

(2) $$\;f_{NE}=\frac{w_{r}-w_{s}\times \left[V_{0}-V_{r}\right]}{w_{b}\times f_{i}\times V_{r}}\times 100$$

where *w_r_* is the weight of non-extracted PHA. Then, the total recovered PHA is *f_TOT_* = *f_E_* + *f_NE_*

**Figure 1:**
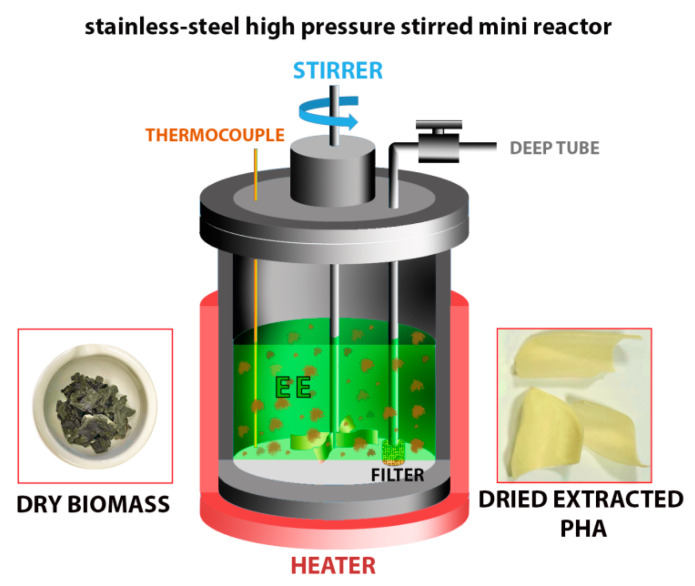
Scheme of the employed stainless-steel high pressure stirred mini reactor, original dry biomass and dried extracted PHA.

A scheme of the employed stainless-steel high pressure stirred mini reactor, original dry biomass and dried extracted PHA is reported in Figure 1.

### 2.4. Characterization Methods

The PHA content in the biomass, the purity and comonomer composition (3HB mol%) of all the extracted samples were evaluated by the gas-chromatographic method according to Braunegg et al. [41]. Approximately 3.5 mg of dried biomass were suspended in 2 mL of acidified methanol solution (at 3% *v*/*v* H_2_SO_4_) containing benzoic acid (at 0.005% *w*/*v*) as internal standard and 1 mL of chloroform in a screw-capped test tube. Then, an acid-catalyzed methanolysis of the PHA occurred and the released methyl esters were quantified by gas-chromatography (GC-FID Perkin Elmer 8410). The relative abundance of 3HB and 3HV comonomers was determined using a commercial P(3HB-*co*-3HV) copolymer with a 3HV content of 5 wt% (Sigma-Aldrich, Milan, Italy) as a reference standard.

The viscosity average molecular weight (*M_v_*) of E-PHA and NE-PHA samples was determined by dilute solution viscosimetry in chloroform at 30 °C. The polymer intrinsic viscosity ([*η*]) was related to *M_v_* by Mark–Houwink Equation (3):

(3) $$\left[\eta \right]=k\times M_{v}^{a}$$

In actuality, the employed values of *k* = 7.7 × 10^−5^ and α = 0.82, suggested by Marchessault et al. [42], were reported for P(3HB) homopolymer. Nevertheless, for comparison purposes, the *M_v_* values of P(3HB-*co*-3HV) copolymer were given hereinafter, taking into account the nearly equal composition and the low 3HV content of PHAs in both biomasses.

All the extracted samples were characterized by FT-IR spectroscopy in attenuated total reflection mode (ATR) by using a Thermo Nicolet 6700 instrument (Thermo Scientific, Waltham MA, USA), equipped with a Golden Gate diamond single reflection device (Specac LTD, Orpington, UK). The spectra were collected co-adding 200 scans at a resolution of 4 cm^−1^ in the range 4000–650 cm^−1^.

The extracted samples with EA at 150 °C were analyzed by 1H-NMR spectroscopy by a Bruker AVANCE III spectrometer (Bruker BioSpin, Karlsruhe, Germany), equipped with a Bruker multinuclear z-gradient inverse probe-head operating at the proton frequency of 400.13 MHz. The sample was solubilized in deuterated chloroform and the 1H spectra were acquired at 298 K employing a spectral width of 15 ppm (6009.13 Hz), 64k data points, 32 scans and a relaxation delay of 6.55 s in order to achieve full relaxation for all the sample protons. 

**Figure 2:**
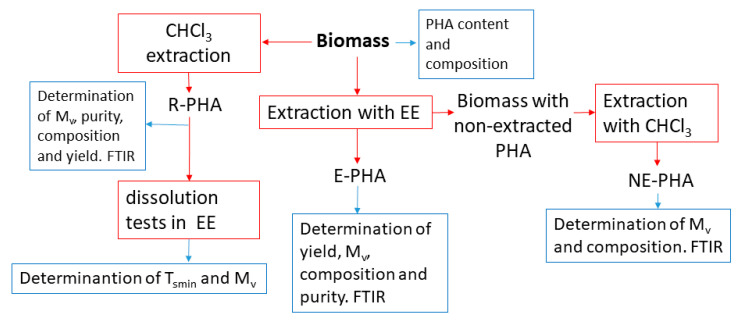
Block diagram of the sample transformations (red lines) and characterizations (blue lines).

The block diagram of sample transformations (red lines) and characterizations (blue lines) is reported in Figure 2.

## 3. Results and Discussion

### 3.1. Screening of Different EEs as PHA-Extraction Solvents

The polymer content and composition obtained by GC analyses of the two biomasses, as well as the viscosity average molecular weight of PHAs extracted in Soxhlet by chloroform (R-PHA1 from Biomass1 and R-PHA2 from Biomass2), are reported in Table 1.

As expected, the chloroform extraction led to a high recovery yield (96 wt%) and both R-PHAs showed high purity (98 wt%) and the same composition found by GC analysis of biomasses.

PHAs in biomasses are exclusively P(3HB-*co*-3HV) copolymers with similar chemical compositions. The high 3HB monomer content was due to the fermented feedstock composition; in fact, the molar fraction of acids containing an odd number of carbon atoms (mainly propionic and valeric, precursors of HV formation) compared to total VFA was quite low and the VFA distribution was strongly oriented to the predominance of acids with even number of C-atoms (mainly acetic and butyric; 0.15–0.17 mol/mol) [10]. This parameter was already used to characterize VFA distribution in complex mixtures and, in turn, to predict the chemical composition of PHA.

The different *M_v_* of the two R-PHA samples is likely due to the different stabilization methods [43,44]. In fact, it has been previously observed how the thermal treatment, used for the stabilization of Biomass1, led to a molecular weight reduction, due to partial polymer hydrolysis favored by high temperature.

At room temperature, EEs do not solubilize the P(3HB-*co*-3HV) and the complete dissolution occurred at a temperature above (EA, EP) or close (EB) to the boiling points of the liquids. In Table 1, the minimum solubilization temperature (T_smin_) of R-PHA1 in the tested EEs and R-PHA2 in EA are reported. For sake of comparison, T_smin_ in EA of the commercial Biomer, a poly(3-hydroxybutyrate) homopolymer (P3HB), was also found. The dissolution tests of R-PHA1 showed that the shorter is the EE acylic residue the lower is the T_smin_ and that the EA solubilized R-PHA2 and Biomer at a higher temperature than R-PHA1, presumably because of the higher molecular weight of these polymers. 

According to the preliminary dissolution tests, the PHA extraction from Biomass1 was carried out initially with the three EEs at T_E_ equal to T_smin_.

After 1 h, at the end of the extraction, the liquid phase was withdrawn from high pressure stirred mini-reactor by the dip tube at T = T_E_ to avoid physical gel formation and to favor the separation of the solution from the solid biomass. In this way, about 92–95 vol% of the initial solvent volume was recovered (*V_r_* in Equations (1) and (2). Then, weight, purity, composition and molecular weight of dry extracted PHA fraction solubilized in EEs (E-PHA1) were measured. Moreover, the fraction (*f_NE_*) and composition of the non-extracted polymer (NE-PHA1) from the biomass were determined by GC. The results of the analyses are displayed in Table 2.

Table 2 highlights that the polymer solubilized from the biomass with the different EEs, after solvent evaporation, shows a high purity and an increase of 3HV comonomeric unit content with respect to those of R-PHA1. Accordingly, in non-extracted NE-PHA, the 3HB mol% value increased. Moreover, it can be observed that all the E-PHA1s, extracted with the three EEs, are characterized by *M_v_* lower than that of the reference sample. Since the extraction by EA gave the best recovery yield and the lowest *M_v_* reduction, further investigation was carried out with this solvent.

### 3.2. Evaluation of PHA Extraction Performances with Ethyl Acetate (EA)

**Figure 3:**
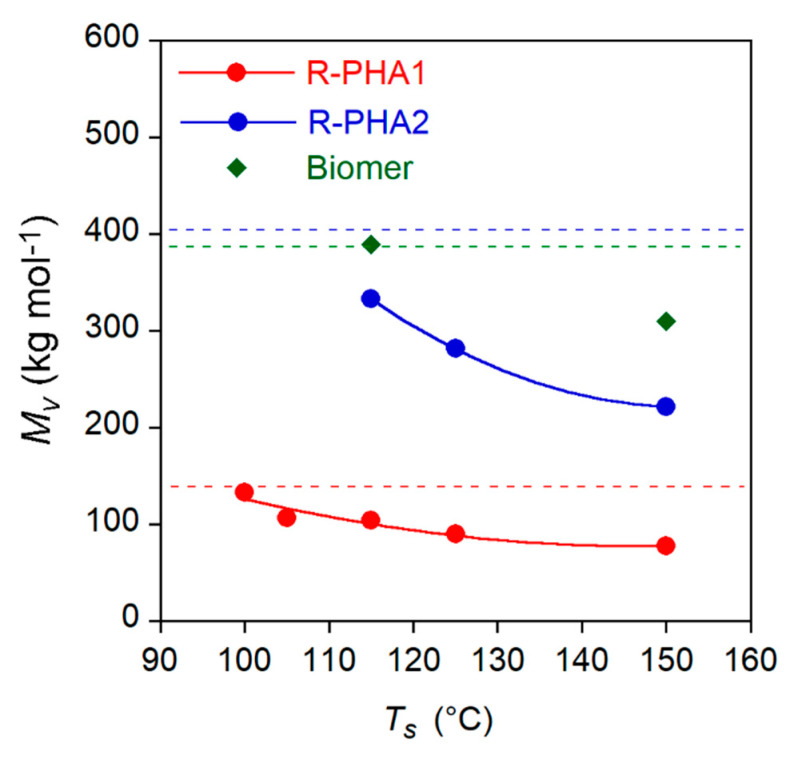
PHA1, R-PHA2 and Biomer viscosimetry average molecular weight (*M_v_*) variation as a function of the dissolution temperature in EA. Dotted lines indicate the *M_v_* values of R-PHAs and of pristine Biomer. Solids curves are a guide for the eyes.

The *M_v_* and composition variation of extracted polymers reported in Table 2 could occur because of selective solubilization of polymer according to its molecular weight and composition and/or of a degradative process taking place at high temperature. Thus, the effect of the temperature on polymer molecular weight was investigated by dissolving the references samples (R-PHA1 and R-PHA2) in EA also at 105 °C < T_s_ < 150 °C. After 1 h, the polymer samples were recovered, dried and the molecular weights determined. In Figure 3, the *M_v_* variation as a function of solubilization temperature T_s_ is reported. For sake of comparison, the experiment was performed also on Biomer at 115 °C and 150 °C.

**Figure 4:**
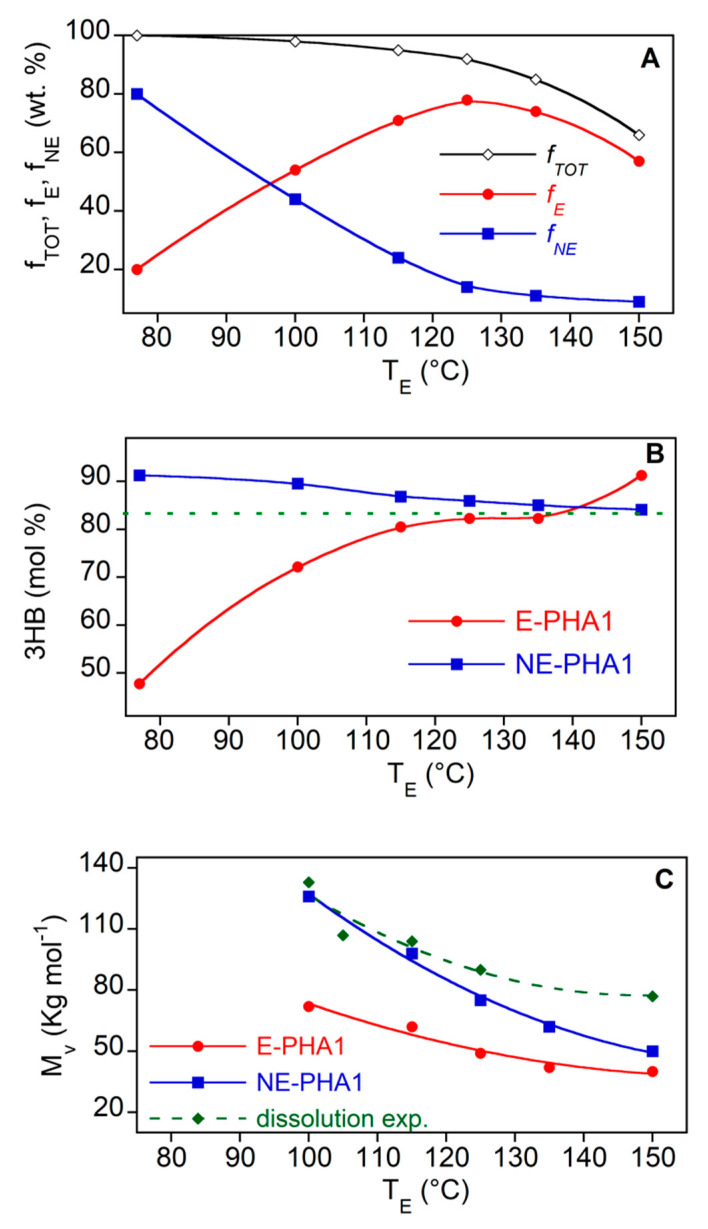
Total (*f_TOT_*), extracted (*f_E_*) and non-extracted (*f_NE_*) PHA fractions (**A**), composition (**B**) and average viscosimetry molecular weight (**C**) of E-PHA1 and NE-PHA1 as a function of extraction temperature T_E_ with EA. The dotted line in (**B**) is the composition of R-PHA1. The green diamond marks in (**C**) are the molecular weight of R-PHA1 after the dissolution experiments already reported in Figure 3. All the lines are guides for the eyes.

Figure 3 shows that the R-PHA dissolution at an increasing temperature brought about a progressive molecular weight reduction up to 44% at 150 °C for both polymers. The homopolymer Biomer seems to be more stable towards degradation phenomena showing a *M_v_* decrease null at 115 °C and of 20% at 150 °C. Therefore, it could be inferred that the polymer in the solution undergoes progressive degradation as the temperature increases. This phenomenon was further investigated to assess the effect of the temperature on the EA extraction of PHA from biomass. Further experiments were carried out by extracting PHA from Biomass1 with EA at the same temperatures of dissolution reported in Figure 3. As in the experiments conducted at T_E_ equal to T_s_, the E-PHA1 and NE-PHA1 fractions (*f_NE_* and *f_E_*, respectively) as well as their compositions and molecular weights were analyzed. The results are reported in Figure 4, where the molecular weight of R-PHA1 obtained in the dissolution experiments, already reported in Figure 3, is displayed for comparison. The results at T_E_ = 77 °C refer to the E-PHA1 extracted in Soxhlet with EA.

Figure 4A shows that the PHA extracted fraction (*f_E_*) increased by increasing temperature up to 125 °C. The decrease at higher temperatures did not reflect the expected increase of the non-extracted polymer fraction (*f_NE_*) remaining in the biomass, leading to a total recovery *f_TOT_* below 100 wt%. Except a small amount of sample lost in the extraction and recovery procedures, this could be attributed to the formation at the highest temperatures of volatile degradation products, as shown later. On the other hand, the purity of the extracted and dried samples was always high, between 90 and 97 wt% (not reported), also without further steps of polymer precipitation with the use of an anti-solvent. Figure 4B shows that the increase of temperature reduced the preferred solubilization of the 3HV rich polymer fraction up to 125–130 °C, where the extracted PHA has a composition similar to that of pristine polymer in biomass. Figure 4C highlights that the PHA fraction solubilized in EA shows a lower *M_v_* than that remaining in the biomass. This lets us infer a preferred degradation of the polymer in solution or a preferred solubilization of shorter chains. Moreover, at T_E_ > 115 °C, the *M_v_* of both E-PHA1 and NE-PHA1 decreases at values lower than that of R-PHA1, heated at the same temperature (green diamond marks), evidencing an active role of the biomass on the reduction of molecular weight. This evidence could be related to the finding of Kopinke et al. [45], that, in a study on P(3HB) kept at a temperature above the polymer melting point, a decrease of the polymer thermal stability in presence of crude biomass was found. More specifically, in thermal degradation investigation on P(3HB-*co*-3HV), Xiang et al. [46] have suggested that calcium ions traces remaining in the copolymer may accelerate the chain scission reaction. In fact, as shown later, the reported chain scission reaction products occurring above the melting point are the same as those observed for the polymer in EA solution at high temperatures. 

Ultimately, the results of PHA extraction from biomass by using EA showed that the recovery yield *f_E_* increases with temperature up to 125–135 °C, but at the expense of a drastic reduction of the polymer molecular weight.

A second extraction for 1 h further at 125 °C of the already extracted biomass brought about a PHA recovery of about 7 wt%. This nearly corresponds to the polymer fraction solubilized in EA but not withdrawn in the first experiment. Therefore, it can be concluded that longer extraction experiments did not lead to a significant *f_E_* increase.

The extraction experiment with EA at T_smin_ = 115 °C was repeated by employing Biomass2. For sake of comparison, the results of the analyses on the extracted and non-extracted polymer fractions were reported in Table 3 together with those obtained from Biomass1 at T_E_ = T_smin_ (100 °C) and at T_E_ = 115 °C.

As for Biomass1, the extraction from Biomass2 brought about the preferential solubilization of polymer fractions with 3HB content and *M_v_* lower than those of the reference sample. Moreover, an overall reduction of PHA *M_v_* took place in all the experiments.

A slightly higher solubility of 3HV-rich PHA and a decrease of *M_v_* of the extracted PHA was also observed by Samorì et al. in extraction experiments carried out by dimethyl carbonate (DMC) [47]. Moreover, it can be observed that at T_E_ = 115 °C, *f_E_* is lower the higher is the molecular weight of the pristine polymer in the biomass. 

To the best knowledge of authors, the EA or other linear esters as PHA extraction solvents are poorly reported in the literature and no examples are available on EEs use as a solvent for the extraction of PHA derived from MMC. As for the PHA solubility in EA, Terada and Marchessault [48], by a semi-empirical approach based on solubility parameters, found that EA could solubilize amorphous P(3HB) but not the polymer in solvent inaccessible crystalline regions. In general, it was observed that short-chain length PHAs, such as P(3HB) homopolymer and P3HB-*co*-3HV copolymers, show lower solubility in non-halogenated hydrocarbon than medium-chain and long-chain-length polymers (mcl-PHA, lcl-PHA). In fact, EA has been successfully used as a solvent for the extraction from biomass of poly(3-hydroxybutyrate-*co*-3-hydroxyhexanoate) (P3HB-*co*-3HHx) containing high levels of HHx (>15 mol%), giving a recovery yield of 99 wt% at 100 °C [28]. More in detail, a comparison between EA and butyl acetate, both applied for extraction from *Ralstonia eutropha* pure cells at 76 wt% of PHA content, indicated EA as the best performing for the recovery yield and that both of them were excellent in the final PHA purity. The same study indicated the necessity to perform the extraction on dried biomass, since the presence of water had a double effect on ester hydrolysis and the reduction of the solvating power. The same extraction by EA of P(3HB-*co*-3HHx) obtained from *Aeromonas hydrophila* 4AK4 has also been employed in industrial-scale production [49]. Conversely, Samorì et al. [29] found a very low recovery (5 wt%) in the extraction of P3HB with ethyl acetate at 80 °C. Similarly, the Soxhlet extraction of P(3HB) with EA has been reported to give a low recovery yield of 29 wt% [50]. However, the unexpected high solubility of high molecular weight PHB (1 × 10^6^ g mol^−1^) in EA at temperatures below the boiling point was found by Aramvash et al. [51], who extracted the polymer from wet biomass with surprisingly high yields. Additionally, Gahlawat and Kumar Soni [52] found a good recovery yield in the extraction of P(3HB-*co*-3HV) at high 3HV content (24.6% mol/mol) with EA, on a pure single strain (*Cupriavidus necator* DSM 545). This study reported a recovery yield of 96 wt%, and a purity of 93% by using EA at 100 °C. They also observed that the extraction brought about a reduction of the pristine molecular weight of PHA in the biomass, from 150.5 to 125 kg mol^−1^.

However, the different results reported in the literature could depend on different aspects such as the PHA composition and the culture type. With specific reference to the latter, it has been generally observed that PHA extraction from MMCs is more challenging in comparison to single strain cultures [17].

A possible extraction of PHA at a temperature above the boiling point with non-solvent or poor-solvent at room temperature, among which EEs are included, has been reported in patents but without indication on yields and possible effects on the composition, chemical or physical–chemical properties of the extracted polymer [21,53].

### 3.3. FT-IR and 1H-NMR Analysis

**Figure 5:**
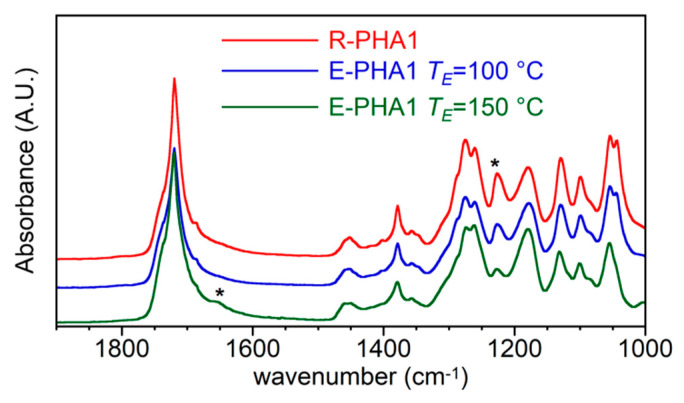
FTIR spectra of R-PHA1 and E-PHA1 extracted with AE at 100 °C and 150 °C.

In order to investigate the cause of the molecular weight reduction of E-PHA, mainly occurring at the higher extraction temperatures, the FT-IR spectra of the extracted samples were compared to those of relevant R-PHAs. In Figure 5, the spectra of R-PHA1 and E-PHA1 extracted at 100 °C as well as 150 °C are reported as an example.

No marked differences can be observed comparing the spectra of R-PHA1 and E-PHA1 extracted at 100 °C, which showed the expected bands of P(3HB-*co*-3HV) copolymer, where the strong band at 1223 cm^−1^, due to C–O–C stretching regular chain conformation, is indicative of high crystallinity. On the other hand, the sample extracted at 150 °C showed a clear hump at 1660 cm^−1^, which can be attributed to a C=C stretching, reported as a possible degradation product located at one terminal of the polymer chain [54], and a drastic decrease of the band at 1223 cm^−1^, which was a sign of the predominant amorphous feature of the polymer. 

**Figure 6:**
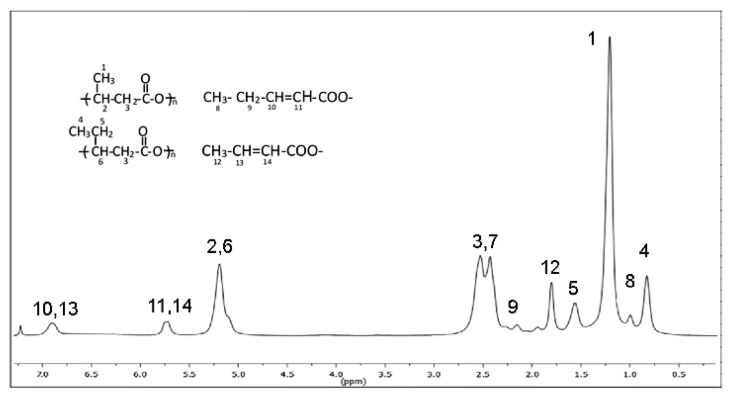
1HNMR of PHA extracted at 150 °C for 1 h and assignments of the peaks.

Then, a further study on the degradation process and the macromolecule scission mechanism was carried out by analyzing the sample extracted at 150 °C by 1H-NMR spectroscopy. The spectrum and the assignments of the signals are reported in Figure 6.

Besides the typical features of the copolymer P(3HB-*co*-3HV), the spectrum showed new signals assigned to unsaturated crotonate terminal groups. They were due to the random chain scission occurring through a β-elimination and α-deprotonation mechanism, according to reported studies on thermal degradation of PHA above the melting temperature [46,55]. Then, the decrease of total polymer recovery (E-PHA1 plus NE-PHA1) recorded at temperatures higher than 125 °C (Figure 4A) can be attributed to the scission of the terminal repeating units which produced volatile low molecular weight product. The absence of peaks of acetate or ethoxy protons lets us exclude that molecular weight reduction was caused by possible transesterification between EA and PHA favored by high temperature. The absence of the C=C stretching absorption in the sample extracted at T_E_ < 130 °C was due to the low concentration of terminal crotonic groups because of the higher polymer molecular weight. However, it can be presumed that the chain scission follows the same reaction mechanism also at T_E_ < 150 °C.

## 4. Conclusions

The present study showed that the extraction of P(3HB-*co*-3HV) copolymers from the biomass by the selected three ethylic esters brings about a polymer with high purity and that the extracted polymer composition, recovery yield and molecular weight depend on the extraction conditions as well as on the MW of pristine polymer in the biomass. The preliminary dissolution tests carried out on reference PHA, extracted from biomass with chloroform, showed that:

- ethyl acetate is the best solvent because it dissolves the copolymer at a temperature lower than ethyl propionate and ethyl butyrate;
- by increasing the temperature from 100 °C to 150 °C, the PHA dissolved in ethyl acetate underwent a progressive reduction of its molecular weight.

The results of the extraction of PHA from biomasses showed that:

- the higher the molecular weight of the polymer in the biomass, the lower the recovery yield;
- at the minimum dissolution temperature, ethyl acetate gave recovery yields higher than the other ethylic esters, and that it preferentially extracts the copolymer fraction richer in 3HV comonomer and with the lower molecular weight;
- by increasing the extraction temperature from 100 °C to 130 °C, the recovery yield increased from about 50 wt% to 80 wt% and the composition of the extracted polymer approached that of the reference sample;
- by increasing the extraction temperature up to 150 °C, a progressive reduction of molecular weight of the extracted polymer and of the polymer fraction remaining in the biomass occurred;
- the purity of the samples extracted with ethyl acetate was always very high, between 90 and 97 wt%, without the need for further purification by anti-solvent precipitation.

FTIR and 1H-NMR analyses, carried out on a PHA sample extracted by the harshest condition (150 °C), showed that the chain scission occurred with the formation of crotonic groups at the polymer ends, through the same mechanism of the PHA thermal decomposition above its melting point.

In conclusion, the use of ethylic esters of VFA could be an attractive method to extract PHA when the use of safe and non-toxic solvents is mandatory, particularly in the case where their synthesis is included in the virtuous cycle of biorefinery. Moreover, the results highlighted that it is possible to choose the proper extraction condition to maximize the recovery yield at the expense of polymer fractionation and degradation at high temperatures or use mild conditions to maintain the original properties of polymer in the biomass.

## Figures and Tables

**Table 1:** Content, composition and viscosity average molecular weight of PHAs in the two biomasses and minimum solubilization temperature (T_smin_) of R-PHAs and Biomer in the tested EEs.

Sample	PHA Content in Biomass (wt%)	Composition (3HB mol%)	*M_v_* (kg mol^−1^)	T_smin_ in EA (°C)	T_smin_ in EP (°C)	T_smin_ in EB (°C)
Biomass1	56	83	–	–	–	–
Biomass2	62	89	–	–	–	–
R-PHA 1	–	83	139 ± 3	100	115	120
R-PHA 2	–	89	405 ± 5	115	–	–
BIOMER	–	100	390 ± 10	115	–	–

**Table 2:** Characterization of the extracted (E-PHA1) and of the non-extracted PHA (NE-PHA1) from Biomass1 with the three EEs.

	E-PHA1				NE-PHA1	
Solvent	*f_E_* (wt%)	Composition (3HB mol%)	Purity (wt%)	*M_v_* (kg·mol^−1^)	*f_NE_* (wt%)	Composition (3HB mol%)
EA at 100 °C	54	72	97	72	44	89
EP at 115 °C	45	74	100	38	52	88
EB at 120 °C	32	57	84	29	62	90

**Table 3:** Summary of recovered fractions (*f_TOT_*, *f_E_*, *f_NE_*), chemical composition and molecular weight of extracted and non-extracted PHA from Biomass1 and Biomass2 with EA.

Sample	Recovered Fractions (wt%)			Composition (3HB mol%)			*M_v_* (kg·mol^−1^)			
	*f_TOT_*	*f_E_*	*f_NE_*	R-PHA	E-PHA	NE-PHA	R-PHA	R-PHA T_E_ = T_s_	E-PHA	NE-PHA
Biomass1 T_E_ = 100 °C	98	54	44	83	69	88	139	133	72	126
Biomass1 T_E_ = 115 °C	94	71	23	83	78	85	139	104	62	98
Biomass2 T_E_ = 115 °C	92	66	25	89	84	92	405	333	236	358

## Abbreviations

3HB: 3-hydroxybutyrate comonomeric unit; 3HV, 3-hydroxyvalerate comonomeric unit; EA, ethyl acetate; EB, ethyl butyrate; EEs, ethylic esters of VFA; EP, ethyl propionate; E-PHA, extracted PHA; *f_E_*, extracted PHA fraction; *f_NE_*, non-extracted PHA fraction; GC, gas chromatography; MMC, Mixed microbial culture; *M_v_*, viscosity average molecular weight; MW, molecular weight; NE-PHA, non-extracted PHA; OFMSW, organic fraction of municipal solid waste; PHA, Poly(hydroxyalkanoate); P(3HB), Poly(3-hydroxybutyrate) homopolymer; P(3HB-*co*-3HV), Poly(3-hydroxybutyrate-*co*-3-hydroxyvalerate) copolymer; R-PHA, reference PHA extracted with chloroform; SS, sewage sludge; T_E_, extraction temperature; T_s_, solubilisation temperature; T_smin_, minimum solubilisation temperature; VFA, volatile fatty acid; WAS, waste activated sludge.
